# Editorial: Cellular heterogeneity in plants

**DOI:** 10.3389/fpls.2024.1417460

**Published:** 2024-04-23

**Authors:** Kengo Morohashi, Amir H. Ahkami, Jeremy E. Coate, Marc Libault

**Affiliations:** ^1^ Faculty of Science and Technology, Department of Applied Chemistry and Bioscience. Chitose Institute of Science and Technology, Chitose, Japan; ^2^ Environmental Molecular Sciences Laboratory (EMSL), Pacific Northwest National Laboratory (PNNL), Richland, WA, United States; ^3^ Department of Biology, Reed College, Portland, OR, United States; ^4^ Division of Plant Science and Technology, College of Agriculture, Food, and Natural Resources, Interdisciplinary Plant Group of Missouri-Columbia, University of Missouri-Columbia, Columbia, MO, United States

**Keywords:** cellular heterogeneity, cell types, cell states, development, biotic and abiotic stress

Multicellular eukaryotic organisms, such as plants, consist of various cell types. Despite possessing the same genetic information, each cell exhibits distinct utilization of this information, resulting in the development of unique molecular, physiological, and morphological properties as well as cellular heterogeneity within the organism. This cellular heterogeneity is needed to support plant development and adaptation to environmental changes. Identifying the mechanisms responsible for the differentiation of distinct cell types and precisely characterizing the molecular, biochemical, biophysical, and morphological characteristics of each cell type in various plant species remains a significant objective for plant scientists. Despite the biological importance of these cellular attributes, they have not been adequately described. In this Frontiers Research Topic, “*Cellular heterogeneity in plants*,” various scientific papers provide valuable new insights into the causes and consequences of cellular heterogeneity in plants.

## Cellular heterogeneity in roots

Roots function to intake water and nutrients and to anchor plants in the soil. Since they attach directly to soils, roots are exposed to various abiotic and abiotic stresses. How different cell types in the root respond transcriptionally to various stresses is an area of active research. Suzuki et al. reported a key gene, *PUCHI*, that controls Arabidopsis root-nematode interactions. Parasitic root-knot nematodes alter the vascular tissue of the host plant into permanent feeding cells, which are giant cells (GCs), to extract nutrients from the host plant. GCs are multinucleated cells that exhibit metabolic activity and possess unique cell wall structures. However, the precise genetic mechanisms governing their formation remain largely unexplored. The authors demonstrate the role of the *Arabidopsis thaliana* transcription factor *PUCHI* in gall development upon root-knot nematode infection. *PUCHI* expression was found to be induced in early developing galls, implying its importance in gall formation. In addition, 3D reconstructions of *puchi* GCs revealed irregular shapes with noticeable cell surface protrusions and folds. Strikingly, the loss-of-function mutant of *3-KETOACYL-COA SYNTHASE 1* showed similar changes in GC morphology and cell wall defects to the *puchi* mutant, suggesting that *PUCHI* may regulate GC development through very long-chain fatty acid synthesis.

The root system also includes nodules, a plant organ where plant-bacteria symbiosis occurs, notably in legumes. For instance, soybeans interact with nitrogen-fixing bacteria (rhizobia) leading to the formation of nodules, in which rhizobia fix and assimilate the atmospheric nitrogen for the plant. Nodules are cellularly heterogeneous organs. To reveal this heterogeneity, Veličković et al. designed a new method that utilizes an enzyme-assisted matrix-assisted laser desorption/ionization mass spectrometry imaging strategy to visualize the distribution and identity of *N*-glycans in soybean root nodules at the cellular level. The authors show varying *N*-glycan profiles in soybean root nodules infected with wild type or mutant rhizobia that are inefficient in fixing atmospheric nitrogen. They found that the majority of complex *N*-glycan structures, including those with Lewis-a epitopes, were more prevalent in the nodules infected by the mutant bacterial strain. Moreover, the authors performed proteomic analysis, revealing that these glycans originated from proteins involved in maintaining redox balance and in *N*-glycan and phenylpropanoid biosynthesis.

## Cellular heterogeneity in development

The vascular system is composed of phloem that transports and distributes organic nutrients and xylem that transports water throughout a plant’s body. In addition to the phloem and xylem, the vascular cambium arises between the primary xylem and phloem. The vascular cambium, a plant stem cell niche, drives radial plant growth by producing secondary xylem inward and secondary phloem outward. The vascular cambium, with its bidirectional characteristics and developmental plasticity, is a prime example for studying the principles of tissue patterning. The mini review by Haas et al. proposes two possible scenarios describing the cell fate decisions of stem cell daughters into xylem and phloem progenitors based on the current knowledge of gene regulatory networks and cellular environments. The two scenarios discussed here may not be mutually exclusive. In the first scenario, the central cambium domain contains cambium stem cells (CSCs) that maintain a stable state over time. In contrast, the second scenario involves a heterogeneous mix of cells with fluctuating states, which are both CSC- and xylem- or phloem-prone.

In addition to the cells in the shoot and root apical meristems, dedifferentiated cells (or calli) facilitate somatic embryogenesis, which involves the transformation of a plant somatic cell into a totipotent embryonic stem cell capable of developing into a fully grown plant. Sahara et al., reveal the transcriptional heterogeneity in palm embryonic cells. Oil palm tissue culture provides elite oil palms with desirable traits through micropropagation. This is commonly performed via somatic embryogenesis, although the success rate is relatively low. RNA-Seq was applied to high- and low-embryogenic ortets of oil palm varieties based on somatic embryos. This study highlights the differences in physiology between high- and low-embryogenic roots, which may impact their ability to undergo somatic embryogenesis.

## Cellular heterogeneity and abiotic stress

Using spatial metabolomics approaches, Balasubramanian et al., found novel distinct drought-responsive metabolic shifts in two major leaf cell types in *Populus*, *i.e.*, palisade and vascular cells. This provides a better understanding on how different plant cell types work together to respond to environmental changes. Understanding spatiotemporal differences in metabolic responses to abiotic stresses will help engineer targeted pathways in specific cell types, potentially achieving maximum productivity under suboptimal conditions and avoiding any pleiotropic effects on plant growth and development (Balasubramanian et al).

## Cellular heterogeneity and cell types

So far in this editorial, we describe the molecular heterogeneity between various cell types. However, through recent advances in single-cell technology, the scientific community has faced a more fundamental yet difficult question: what is a cell type? Doyle and Amini et al., offer opinions on this subject, opinions that might change based on future discoveries. The unprecedent resolution brought by single-cell RNA-seq has simultaneously revealed heterogeneity among cells previously considered homogenous and blurred the lines between populations of cells thought to be distinct. As a result, it is challenging to clearly distinguish cell types and this has led some to argue there are no cell types, but only cell states. Amini et al. argue that transcriptomic definitions have tremendous utility, but highlight other definitions, such as those based on function, lineage or evolution, and suggest these or other approaches to defining cell types will ultimately move the field forward. Doyle illustrates how the challenges cell biologists face defining and delineating cell types parallel the challenges systematists face in defining and delineating species, and highlights areas in which each field could learn from the other.

## Author contributions

KM: Writing – original draft, Writing – review & editing. AA: Writing – review & editing. JC: Writing – review & editing. ML: Writing – review & editing.

